# Limpet Shells from the Aterian Level 8 of El Harhoura 2 Cave (Témara, Morocco): Preservation State of Crossed-Foliated Layers

**DOI:** 10.1371/journal.pone.0137162

**Published:** 2015-09-16

**Authors:** Julius Nouet, Corinne Chevallard, Bastien Farre, Gernot Nehrke, Emilie Campmas, Emmanuelle Stoetzel, Mohamed Abdeljalil El Hajraoui, Roland Nespoulet

**Affiliations:** 1 Université Paris Sud, CNRS UMR GEOPS 8148, bâtiment 504, campus universitaire, 91405 Orsay cedex, France; 2 CEA, CNRS IRAMIS, UMR SIS2M 3299, LIONS, CEA-Saclay, F- 91191 Gif-sur-Yvette, France; 3 ISTO, CNRS UMR 7327, 1A rue de la Férolerie 45071 Orléans cedex 2, France; 4 Alfred Wegener Institute for Polar and Marine Research, Am Handelshafen 12, 27570 Bremerhaven, Germany; 5 Université Toulouse Jean Jaurès, CNRS UMR TRACES 5608, Maison de la Recherche, 5 allée Antonio Machado, 31058 Toulouse, France; 6 Institut National des Sciences de l’Archéologie et du Patrimoine, angle rues 5 et 7 Rabat Instituts, Madinat Al Irfane, Rabat Hay Riyad, Morocco; 7 Muséum National d’Histoire Naturelle, Département de Préhistoire, CNRS UMR 7194, Musée de l’Homme, bureau 345, Paris, France; University of Oxford, UNITED KINGDOM

## Abstract

The exploitation of mollusks by the first anatomically modern humans is a central question for archaeologists. This paper focuses on level 8 (dated around ∼ 100 ka BP) of El Harhoura 2 Cave, located along the coastline in the Rabat-Témara region (Morocco). The large quantity of *Patella* sp. shells found in this level highlights questions regarding their origin and preservation. This study presents an estimation of the preservation status of these shells. We focus here on the diagenetic evolution of both the microstructural patterns and organic components of crossed-foliated shell layers, in order to assess the viability of further investigations based on shell layer minor elements, isotopic or biochemical compositions. The results show that the shells seem to be well conserved, with microstructural patterns preserved down to sub-micrometric scales, and that some organic components are still present *in situ*. But faint taphonomic degradations affecting both mineral and organic components are nonetheless evidenced, such as the disappearance of organic envelopes surrounding crossed-foliated lamellae, combined with a partial recrystallization of the lamellae. Our results provide a solid case-study of the early stages of the diagenetic evolution of crossed-foliated shell layers. Moreover, they highlight the fact that extreme caution must be taken before using fossil shells for palaeoenvironmental or geochronological reconstructions. Without thorough investigation, the alteration patterns illustrated here would easily have gone unnoticed. However, these degradations are liable to bias any proxy based on the elemental, isotopic or biochemical composition of the shells. This study also provides significant data concerning human subsistence behavior: the presence of notches and the good preservation state of limpet shells (no dissolution/recrystallization, no bioerosion and no abrasion/fragmentation aspects) would attest that limpets were gathered alive with tools by Middle Palaeolithic (Aterian) populations in North Africa for consumption.

## Geographical and geological setting

**Fig 1:**
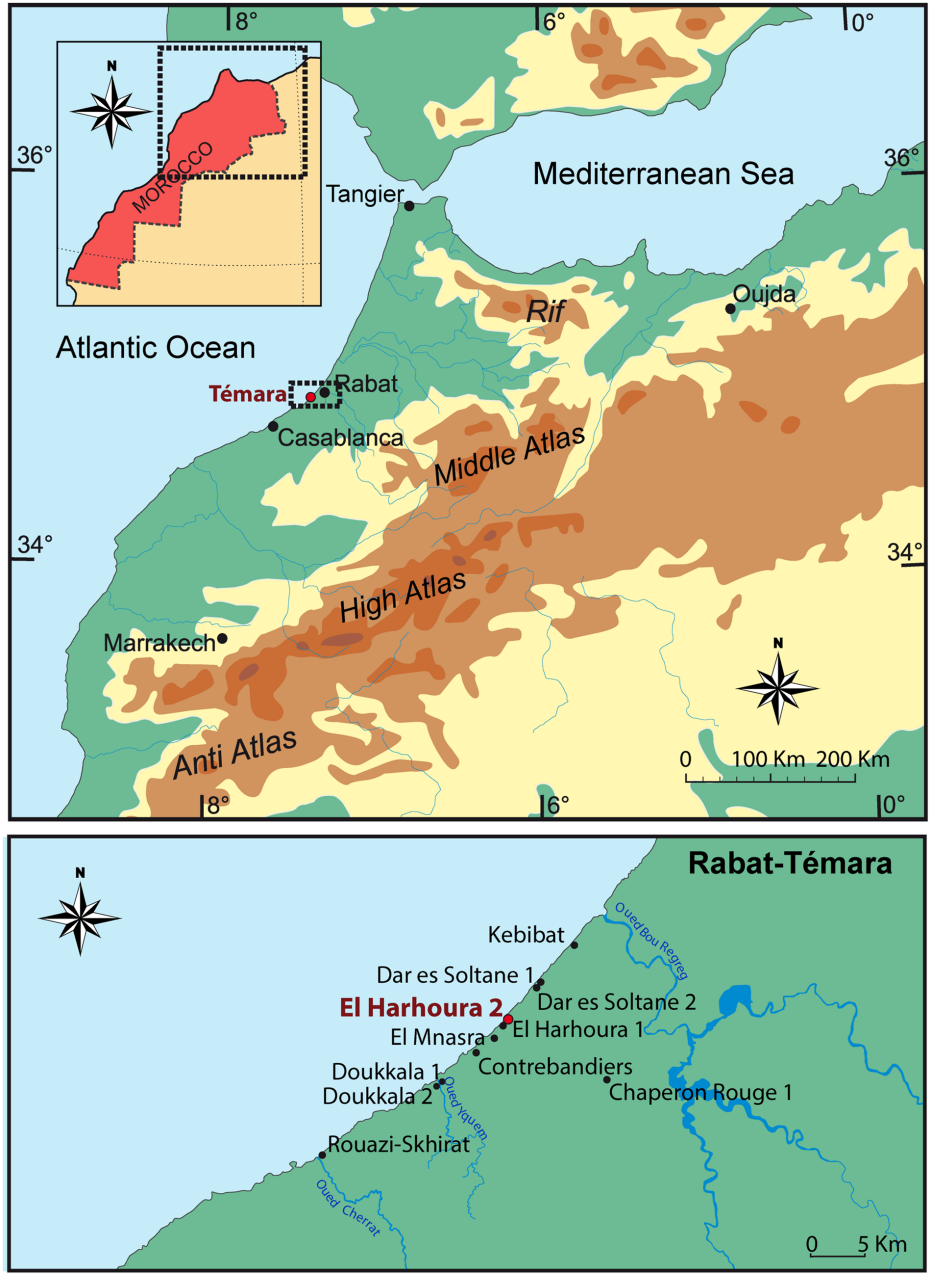
Map of North Morocco and localization of the caves of archaeological interest on the Atlantic coast of Rabat-Témara.

El Harhoura 2 belongs to a network of littoral caves located in the Témara-Rabat area (Fig 1). These caves are mainly known for the first fossil remains attributed to anatomically modern humans (AMH), [1–6] as well as *Nassarius* shell beads [7, 8], bone tools [9–11], and evidence of pigment use [6, 12] associated with the North African Aterian culture (Middle Palaeolithic [MP]/Middle Stone Age [MSA]) [6, 13–18]. This area is therefore important for the debate concerning the emergence of complex early AMH behaviors in North Africa, and particularly in a coastal context.

**Fig 2:**
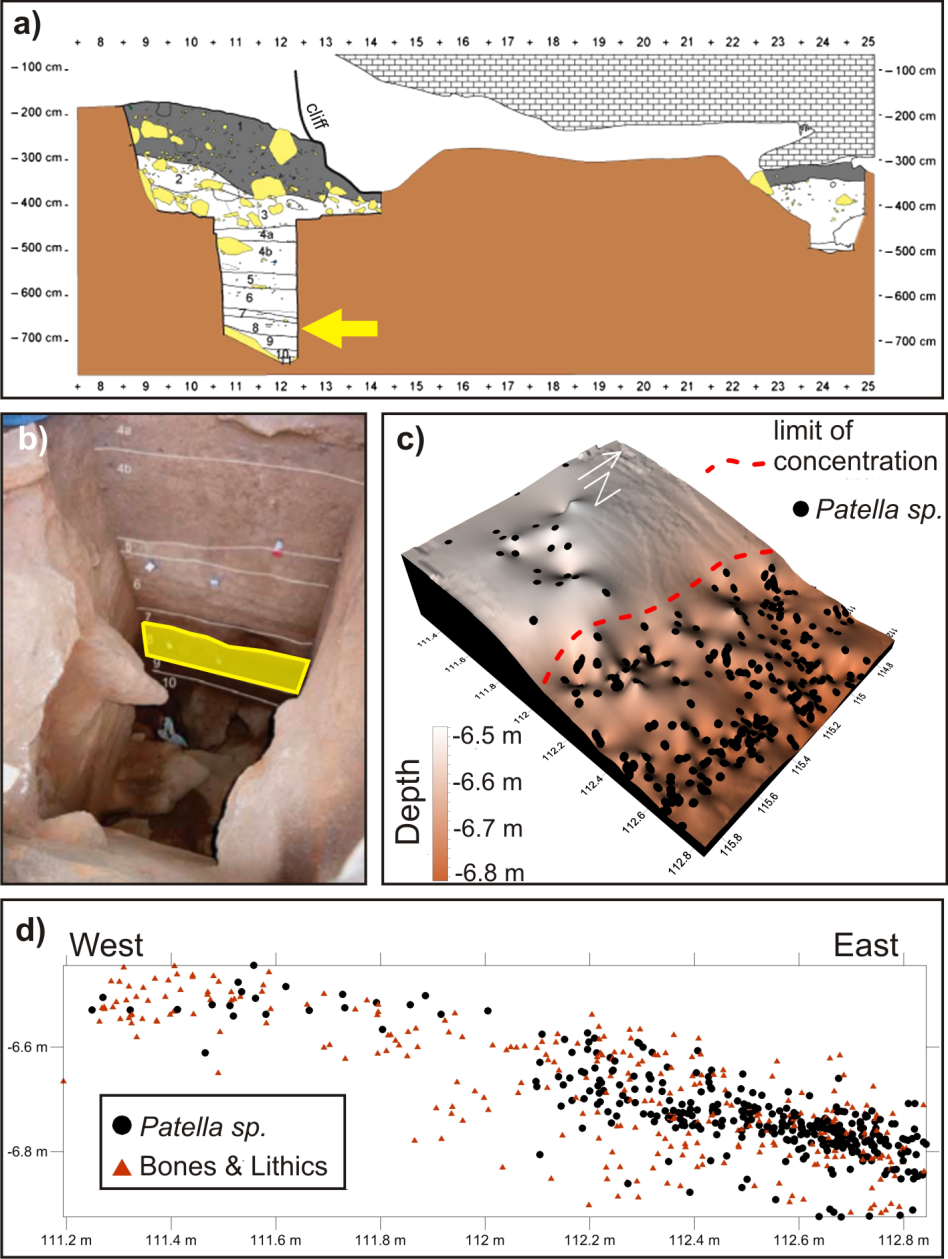
El Harhoura 2 cave. **a)** Stratigraphic section of the cave, showing the front test pit and back excavation. Arrow marks level of interest 8. Level 1, Neolithic; level 2, Upper Paleolithic; levels 3—11, Middle Paleolithic. **b)** Picture of the front drill, with highlighted delimitation of level 8. **c)** Plot of the surface of the section of level 8 excavated during the reconnaissance drill, with the localizations of *Patella* sp. specimens. **d)** East-west profile of the drill at level 8 depth showing the concentration of *Patella* sp. specimens, complete or fragmented bones, and lithic materials.

El Harhoura 2 cave is carved into the base of the Oulijan calcarenite cliff extending from the south of Rabat down to Skhirat, in the vicinity of Témara. This cave was probably formed by water erosion before the marine incursion of isotopic stage 5e (∼ 125 ka BP) and later filled by sediments supplied by the local coastal environment, mostly sandy ridges, during the last glacial period (starting at isotope stage 5e) [19] (Fig 2a). Excavations have provided substantial evidence of human occupations from the MP/MSA (Aterian) to the Neolithic (∼ 120 ka to 6 ka BP) [20–22] with a well-documented archaeological context [6, 19, 23]. These excavations have also provided many large faunal and microfaunal remains, [11, 17, 24–27], lithic industries [6, 28], human remains [29], as well as numerous mollusk shells (snails, mussels, limpets). However the bedrock has not yet been reached by the test pit.

In level 8 (Fig 2b), dated between 92 +11/-9 ka BP (ESR-U/Th method) [22] and 106.7 +/- 6.6 ka BP (OSL method) [21], a large accumulation of Patellidae, or “limpet” shells, was found, deposited in a hollow on the upper surface of the level (Fig 2c) along with numerous faunal/microfaunal bones and lithic material (Fig 2d).

## Introduction

Shell accumulations are found in many MP/MSA archaeological coastal sites in Southern Europe and in Africa [30–36]. These deposits are mainly composed of mollusk species living on intertidal rocky shores and collected by humans. These species are clearly visible, easily collectable [31, 37] and concentrated in clusters in comparison to sandy shores mollusk assemblages (with a 14 times higher macro-invertebrate biomass) [38]. Therefore, it seems that such mollusks represented a significant contribution to the diet of human populations inhabiting coastal areas [39].

These kinds of mollusk shell deposits are widely investigated in relation to palaeoenvironmental reconstructions. Shell isotopic analyses or trace element composition are used as proxies for sea-water temperature or salinity reconstructions [40]. The shells can also be used to trace out harvesting seasons [41, 42], human population densities [43], the exploitation of aquatic resources [44], and the emergence of modern human behaviors [45].

In most cases, mollusk shells cannot be used ¨blindly¨ for palaeoreconstructions or other analyses. For example, geochronological reconstructions are usually considered unreliable when using the mineral component of mollusk shells as potential proxies for dating sedimentary deposits, either by using uranium series [46, 47] or radiocarbon [48]. This led some authors to develop dating methods based on the organic matrix of the shells: such as Demarchi et al. [49], who recently claimed that *Patella* shells might be reliable archives for geochronological reconstructions, using intracrystalline protein diagenesis (IcPD) analysis. More generally, organic components closely associated with the mineral phase down to sub-micrometric scales in mollusk shells [50–52], and frequently preserved in fossil shells, might prove to be useful proxies, but are often undervalued.

All of the above cited investigations must first take into account the biocomposite nature and complex architectural organization of these materials; two points that are still largely underestimated in the literature. The macromolecules incorporated in the mineral phase are widely thought to play an active role in biomineralization [53–56]. In spite of the fact that biocrystallization mechanisms themselves are still largely unknown, it is now recognized that they differ from classical crystal nucleation and growth [55, 57–59]. These uncommon processes can lead to systematic biases for paleo-proxies [60, 61] and complex diagenetic patterns. Additionnally the preservation state of the shells not only depends on local taphonomic conditions, but also varies widely according to microstructural types, mineralogy or the organic composition of individual shell layers. The state of alteration of shell layers should be systematically assessed before starting any other analysis. Unfortunately, this preliminary step is often omitted.

Patelloid gastropod shells provide a good example of microstructural complexity, as they display sophisticated organization, with several layers of various microstructural types and orientations [62]. Among patelloids, the Patellinae sub-family is unique, as its members are the only gastropods displaying the crossed-foliated microstructural type [63]. This microstructure is the calcitic counterpart of the crossed-lamellar microstructure (undoubtedly the most common type in gastropod and bivalve shells) [64] and it composes most of the outer parts of the Patellinae shell. The other layers are mainly aragonite crossed-lamellar. The crossed-foliated microstructure shares a complex 3D architecture similar to that of the crossed-lamellar, but with subtle variations. This microstructure is composed of interlaced units at several orders of magnitude. The largest structural unit (termed first order lamellae) is described as ∼ 80 *μ*m large lamellae showing alternating crystallographic orientation every two lamellae (whereas the mean width of crossed-lamellar first order units is only ∼ 15 *μ*m) [63]. The first order lamellae themselves are composed of ∼ 200 nm thick sheet-like arrangements (termed second order lamellae) of individual rods, termed third order lamellae. The third order rods dip in opposite directions between two consecutive first order lamellae, with a significantly smaller constant angle in crossed-foliated than in crossed-lamellar structures [63, 65]. Here, we will focus on the calcite crossed-foliated microstructural pattern. Calcite is a stable polymorph of Ca-carbonate at room temperature and normal pressure, and calcite layers are therefore less prone to recrystallization and should be better preserved than the slightly less stable aragonite layers.

The aim of this work is to lay the foundations for an estimation of the state of preservation of the crossed-foliated layers of *Patella* shells sampled during excavations at El Harhoura 2 and to discuss their possible consumption by Aterian populations. Indeed, the large quantity of *Patella* sp. shells found in the studied level raises questions regarding their origin and preservation; is the high quantity of limpets in this particular level due to intensive collection and consumption by humans associated with exceptional shell preservation? In order to decipher the taphonomic/diagenetic processes heterogeneously affecting both organic composition and microstructural organization, we will focus on detailed *in situ* characterization techniques using optical, scanning electron or atomic force microscopy, with systematic comparisons to modern *Patella vulgata* specimens, used as a reference for crossed-foliated shell layers features. This preliminary step should ascertain whether these shells can be used for future palaeoenvironmental, chronological or seasonal investigations.

## Materials and methods

### Materials

**Fig 3:**
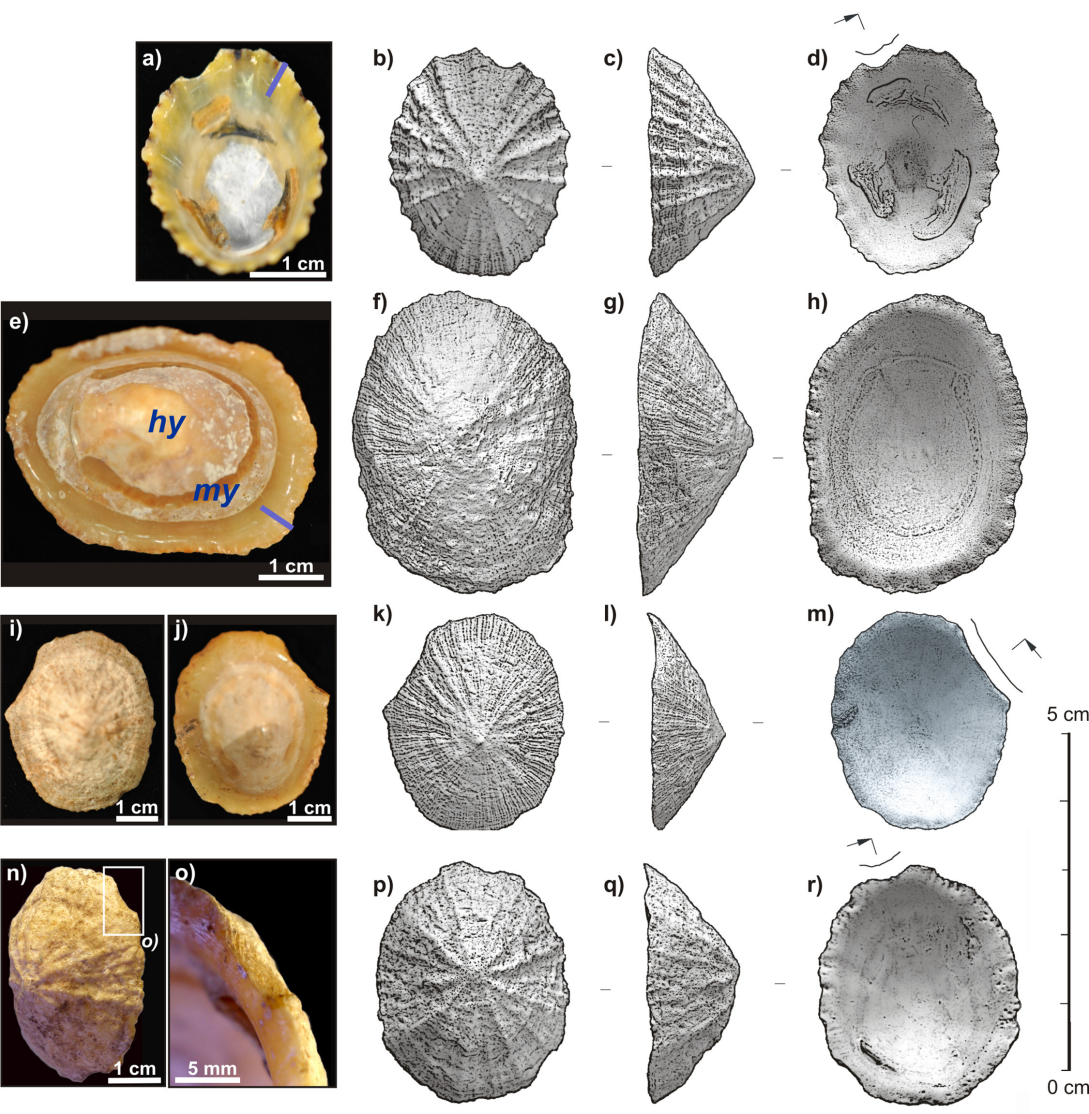
Morphologies of specimens of interest. **a-d)** Modern *Patella vulgata* from the Brittany sea shore. Blue bar: section of m+3 and m+2 layers. Arrow shows the mark left by the knife used to collect the specimen. **e-h)** Unbroken *Patella* sp. from El Harhoura 2 cave (level 8). my: myostracum. hy: hypostracum. Blue bar: section of m+3 and m+2 layers.**i-m)** *Patella* sp. from El Harhoura 2 cave (level 8) with a large broken edge (marked by arrow). **n-r)** *Patella* sp. from El Harhoura 2 cave (level 8) showing a small imprint on its edge (marked by arrow).

The fossil *Patella* specimens come from level 8 of El Harhoura 2 Cave, Témara, Morocco, from the test pit located in the current cave entrance (permit for field studies delivered by A. Akerraz, head of the Institut National des Sciences de l’Archéologie et du Patrimoine du Maroc—Ministère de la Culture—to the two co-directors of the archaeological mission ¨El-Harhoura-Témara¨, R. Nespoulet and M.A. El Hajraoui: the fossils are labeled O12-T1 to O12-T6 and are stored in the GEOPS paleontological collection—ID RSJTL). All the shells were rinsed with deionized water and softly sonicated for 20 s to remove the sediment. For comparative purposes, an attempt was made to collect living *Patella* specimens from the seashore of Témara. However, the very thin shells were heavily degraded by microborers activity, to such an extent that outer crossed-foliated layers were often missing. More robust modern specimens of *Patella vulgata* (Linnaeus, 1758) (Patellogastropoda, Patellidae) were therefore collected live from the sea shore at St Cast, Brittany, France (48°38’19”N, 2°15’09”O—no specific permission was required, as the species is not protected or endangered). The soft tissues of the animals were removed and the shells were rinsed with deionized water. Radial sections of the crossed-foliated outer layers were cut at the border of fossil and modern shells (Fig 3a and 3e).

### Methods

Following the observation of the external morphology of the shells, we chose to combine light, electron and atomic force microscopies in order to investigate both the mineral and the organic components of the shells, at various scales. Polarized Light Microscopy (PLM) was used to examine structural and microstructural patterns and crystallographic features. Confocal and non-confocal Fluorescence Microscopy was performed to inspect the distribution of organic compounds; we mainly used the natural fluorescence of the samples, but some colorations were also tested using a stain specific to a few molecules (acridine orange). Confocal Raman Microscopy (CRM) was used to identify some organic molecules, using their specific Raman signature, and their distribution was mapped. CRM was also used to confirm the mineral phase and characterize some crystallographic features. Scanning Electron Microscopy (SEM) was used to characterize very fine microstructural patterns, completed by Electron Microprobe to map element distribution and Atomic Force Microscopy (AFM) to characterize mechanical properties.

#### Light Microscopy

Polarized Light Microscopy: Standard thin sections (∼ 30 *μ*m) were prepared using HERMES water grinding papers (successively P1200/P2500 and P4000 grain sizes) and observed in transmission using polarized light, as well as polarized and analyzed light (using crossed polarizers).

Fluorescence Microscopy: For epifluorescence observations, sample sections were polished using P1200, P2500, and P4000 HERMES water grinding papers, 3 and 1 *μ*m Buehler diamond polycrystalline suspensions, and aluminum oxide suspension (∼ 300 nm size). Surfaces were carefully rinsed with deionized water. Two samples were then softly etched for 10 s in a formic acid solution (0.1 wt.%) with glutaraldehyde (5 wt.%), and colored with acridine orange for 30 min (20 mg mixed in 95 ml of water and 5 ml of methanol). Observations were made using a Zeiss Universal microscope equipped with x6 (NA = 0.2) and x25 (NA = 0.6) fluorite objectives, a mercury lamp, a 365 nm excitation filter (UV), and a 420 high-pass emission filter. For observation of acridine-stained samples, a 435 nm excitation filter (blue) and a 510 nm high-pass emission filter were used, as the emission wavelength of this fluorophore varies from green to red depending on its environment (see details in the discussion section).

Confocal Laser Scanning Microscopy: Samples were prepared as for epifluorescence microscopy. Observations were carried out on an Olympus FV-1000 inverted confocal microscope (located at LIONS laboratory, CEA-Saclay, France). The sample surface was excited with a green He-Ne laser (*λ* = 488 nm) and emission light was collected through a band pass BA 560 IF filter. 20x (Olympus UPLSAPO, NA = 0.75) and 40x (Olympus UPLFLN, NA = 0.75) objectives were used, with a confocal aperture set at 80 and 130 *μ*m, respectively. All scans were focused ∼ 2 *μ*m under the sample surface, to avoid any potential surface contamination or polishing remains.

Raman mapping was conducted using a WITec alpha300R (WITec GmbH, Germany) confocal Raman microscope. Scans were performed using a piezoelectric scanner table with a maximum scan range of 200*μ*m × 200*μ*m and a minimum lateral step size of 4 nm and a vertical step size of 0.5 nm. The sample surface was excited with a green diode laser (*λ* = 532 nm), and the Raman signal was collected with a dedicated ultra-high throughput spectrometer (UHTS 300, WITec, Germany), using a grating, 600/mm and 500 nm blaze. A Nikon 100x (NA 0.9) objective was used. The spectral analysis and imaging processing was performed using the WITecProject software (version 2.04, WITec GmbH, Germany). Peak positions were determined using the “Mulipeak Fitting 2” routine of IGOR Pro using a Gauss shape for the fitting (version 6.11, WaveMetrics, Inc. USA). To identify calcite, we used calcite peak T_*c*_ at 155 cm^−1^ (translation mode) and L_*c*_ at 282 cm^−1^ (librational mode) and the two internal modes (in-plane band *ν*_4_ at 711 cm^−1^ and symmetric stretch *ν*_1_ at 1085 cm^−1^).

#### Scanning Electron Microscopy

For SEM, several freshly broken, unetched radial shell fragments were prepared. A staining assay was conducted by putting one of the fragments in a sealed container next to an OsO_4_ solution (4% vol. in H_2_O) for 1h. This compound is commonly used to stain organic components in TEM preparations. Even though no obvious staining was observed in our case, contact with the vapor was still found to perform very faint etching at a submicrometric scale. Observations were carried out with two microscopes: 1/ a Philips XL30 SEM using a secondary electrons collector (located at IDES UMR 8148). In this case, the SEM was operated at 25 keV and 10 mm working distance and the samples were coated with Au/Pd. 2/ a Carl Zeiss Ultra Plus Field Emission SEM using a secondary electrons collector (located at the mineralogy department of NHM, London). This FEG-SEM was operated at 1 keV and a 4 mm working distance with an aperture of 10 *μ*m. Samples were not coated.

#### Electron Microprobe

Samples were included in epoxy resin, cut, and polished using HERMES water grinding papers (grain size P1200/P2500 to P4000) and Buehler diamond polycrystalline suspensions (3 *μ*m and 1 *μ*m). Surfaces were carefully rinsed with deionized water and then carbon-coated. Ca, Mg, Sr, and S distributions were mapped using wavelength-dispersive spectrometry on a Cameca SX100 electron microprobe (at the Natural History Museum of London, UK). The microprobe was operated at 15 kV accelerating voltage with a 20 nA specimen current.

#### Atomic Force Microscopy

For AFM observations, samples were polished using HERMES water grinding papers (grain size P1200/P2500 to P4000), Buehler diamond polycrystalline suspensions (3 *μ*m and 1 *μ*m), and finally aluminum oxide suspension (300 nm). The obtained surface was very gently cleaned using a diluted formic acid solution (0.1 wt.%) with glutaraldehyde (3 wt.%) for 1 s, to remove pollution or dust generated by polishing. As shown in existing studies [66], no major artifact is induced by such a process. AFM observations were carried out on a Veeco Dimension 3100 operated in tapping mode, in which height and phase error signals are recorded. The phase error signal gives insights into surface properties: the phase lag between the period of oscillation imposed to the cantilever and the recorded period, at each point, is due to interactions between the tip and the sample surface. Strong phase-lag contrasts therefore reveal contrasts in elastic deformation or surface adhesion effects.

## Results

### Morphology

To the naked eye, the fossil shells used in this study seem well preserved and do not present any rounded or polished aspect due to water/sand abrasion. However, all of them are depigmented (Fig 3e, 3i, 3j, 3n and 3o) and brownish in color, like the sediment. Although they are mostly complete, a significant number of fossil shells bear damage on part of the edge (Fig 3i–3r), displaying a typical semi-circular section. In some cases, this damage is quite extensive (Fig 3m), and is more faintly marked in others (Fig 3o). On several specimens, these notches are not present (Fig 3e). Very similar imprints can be observed on modern shells, collected live (Fig 3a–3d), using a knife to detach them from the bedrock. The adhesive capacity of the limpet foot is considerable and the total force required for large limpet detachment can sometimes reach up to 980 N [31]. This force is however significantly lower if the limpet is caught unaware. A harvest obtained using appropriate gathering behavior and tools therefore results in assemblages of shells scratched to varying degrees, very similar to the shell accumulation found at El Harhoura 2.

Fossil shells are quite large and thick (mean width: 42.0 +/- 5.1 mm; mean thickness: 2.3 +/- 0.3 mm) compared to modern specimens (mean width: 36.7 +/- 3.9 mm; mean thickness: 1.8 +/- 0.2 mm). They may be *Patella rustica* (Linnaeus, 1758) or *Patella vulgata* (Linnaeus, 1758). Both these species are currently found along the Atlantic coasts of Morocco [67]. Steele and lvarez-Fernández [32, 68] have identified several species of Patellidae at Contrebandiers Cave, also located at Témara: *Patella caerulea, Patella nigra [safiana], Patella ferruginae, Patella intermedia [depressa], Patella rustica [lusitanica], Patella ulyssiponensis [aspera, tarentina] and Patella vulgata*, the latter being the best represented. Fossil shells will hereinafter be referred to as *Patella* sp.

Following the notation of [63], shell layers are labeled according to their position with respect to the myostracal layer (m+3: outermost layer, etc). The myostracum, composed of spherulitic and prismatic aragonite layers [69], is easy to identify as it forms a typical horseshoe-shape where the ring muscle of the limpet connects to the shell (Fig 3a), the imprint of which is well preserved in fossil shells (Fig 3e and 3j). In Patellinae, the crossed-foliated microstructure is mainly found in m+3 and m+2 layers and occasionally in a very thin internal layer (m-2). Using Feigl coloration [70], the other layers are confirmed to still be aragonitic in all fossil *Patella* sp. shells.

### Elemental compositions and microstructural features

#### Modern *Patella vulgata*

**Fig 4:**
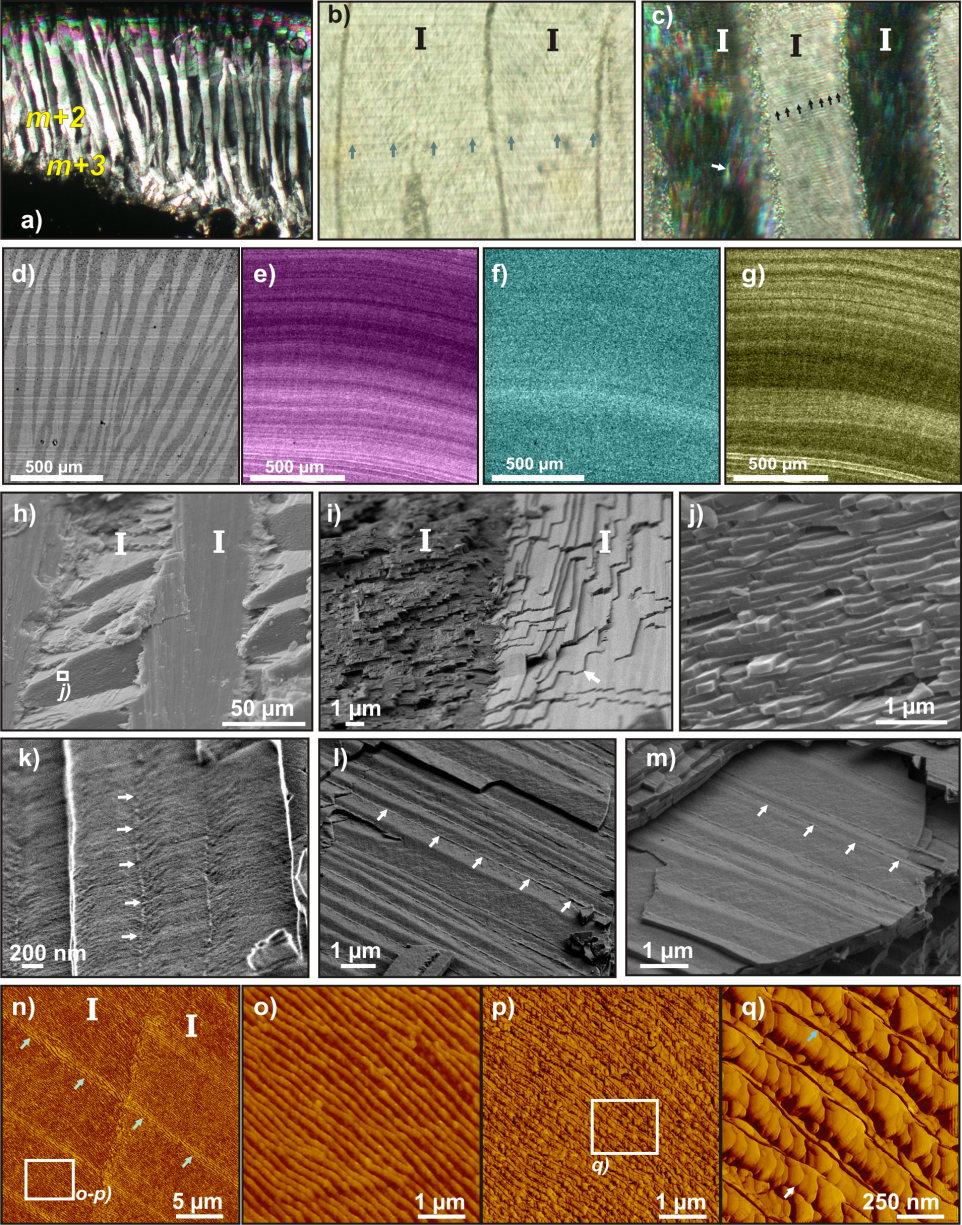
Microstructural organization of modern *Patella vulgata* calcite crossed-foliated outer layers. **a)** PLM view (polarized and analyzed light) of a radial thin section. Outer surface is at bottom. **b)** PLM view (polarized light) showing the growth increments (green arrows) between two consecutive 1^*st*^ order lamellae (I). **c**) PLM view (polarized and analyzed light) of three consecutive 1^*st*^ order lamellae (I), displaying the preserved alternate orientation one lamella on two. 2^*nd*^ order lamellae are visible (black arrows), as well as the individual, faintly disoriented 3^*rd*^order rods (white arrow). d-g) Electron micropobe maps. **d)** Backscattered image of the scanned area, showing the alternate 1^*st*^ order lamellae. **e)** Distribution of Mg content, layered following growth layers. **f)** Distribution of Sr content, displaying very faintly marked growth layers. **g)** Distribution of S content, faintly marking the crossed-foliated structure, strongly marking the growth layering. **h-k)** SEM images of a radial, unetched, freshly broken section. **h)** Several consecutive 1^*st*^ order lamellae (I) (SEM). **i)** Limit between two 1^*st*^ order lamellae (I), showing the change of orientation of its constituting 3^*rd*^order rods (white arrow) or slats (FEG-SEM). **j**) 2^*nd*^ order lamellae, composed of superimposed rows of 3^*rd*^order units (SEM). **k)** Surface view of three consecutive 3^*rd*^order slats within a second order row, separated by faint, punctuated limits (white arrows) and showing an inner texture (FEG-SEM). **l-m)** FEG-SEM images of a radial freshly broken section etched by OsO_4_ vapor, revealing organic membranes that separates each 3^*rd*^order rod (white arrows). **n-q)** AFM scans. **n)** Phase image of the contact between two 1^*st*^ order lamellae. Green arrows mark a growth increment. **o-p)** Height and phase images of 2^*nd*^ order lamellae within a 1^*st*^ order lamella. **q)** Phase image of several 2^*nd*^ order lamellae, separated by a seemingly continuous membrane (blue arrow). Some ovoid sub-units (white arrow) can be seen, constituting the lamellae.

The organization of the shell margin cross-section is well illustrated using PLM (Fig 4a). The M+3 layer is thin and displays a radial crossed-foliated pattern and the m+2 layer makes up the bigger part of the shell, with a very regular concentric crossed-foliated pattern. In polarized light, growth increments are very well marked and regular, and perfectly continuous through consecutive first order lamellae (Fig 4b). The use of crossed polarizers (Fig 4c) allows us to determine the orientation of the first lamellae. The simultaneous extinction of one out of two lamella is evidence that these lamellae present a similar orientation, whereas two consecutive lamellae have distinct orientations (different intensities). Second order lamellae are also clearly visible (black arrows), as well as the individual third order rods (white arrow), when they are faintly disorientated within an extinct first order lamella.

On electron microprobe maps, the distribution of some minor elements strongly marks growth layering, perpendicular to the first order lamellae, which are easily identified in the backscattered electrons image of the scan (Fig 4d). In particular, Mg (Fig 4e) and S content (Fig 4g) display marked contrast, with thin (enriched or depleted) growth bands, whereas Sr content distribution shows much fainter variations (Fig 4f). No correlation can be found between minor element contents in the three distribution maps. In both Mg and S content distribution maps, slight contrast can be observed between consecutive first order lamellae, one out of two lamella, and anti-correlated: the lamellae with higher S content also contain less Mg (and vice-versa). Mn and Ba were also measured but are present in fairly low quantities and homogeneously distributed in the shell, and therefore the maps are not shown here.

SEM views of freshly broken sections illustrate the classical organization of crossed-foliated microstructural type. Between each first order unit (Fig 4h) the third order elongated rods or slats (Fig 4i) dip in opposite directions with a constant angle, and second order lamellae are composed of superimposed rows of third order units (Fig 4j). On a surface view of a second order lamella, a complex and very fine pattern can be distinguished within its third order rods. Moreover, the rods are separated from each other by a faint, very thin, and seemingly punctuated limit (Fig 4k). On the broken section, in contact with OsO_4_ vapor, the very soft alteration of second order lamellae surfaces enhances the mineral/organic contrast, revealing fine sheets separating each third order unit (Fig 4l and 4m).

On AFM views, growth increments are well marked and perfectly continuous between adjacent first order lamellae (Fig 4n). Second order lamellae are visible (Fig 4o and 4p) and are also separated by a thin rim (Fig 4q), displaying a strong contrast in phase image. This contrast would result from a higher interaction between the tip and the sample surface at the border of the lamellae, and highlights the presence of a more visco-elastic material: most probably, organic sheets of meshes. If the delimitation of the third order slats is not so clear, we observed the presence of ovoid sub-units of irregular size and shape and highlighted by faint visco-elasticity contrasts (Fig 4q).

#### Fossil *Patella* sp

**Fig 5:**
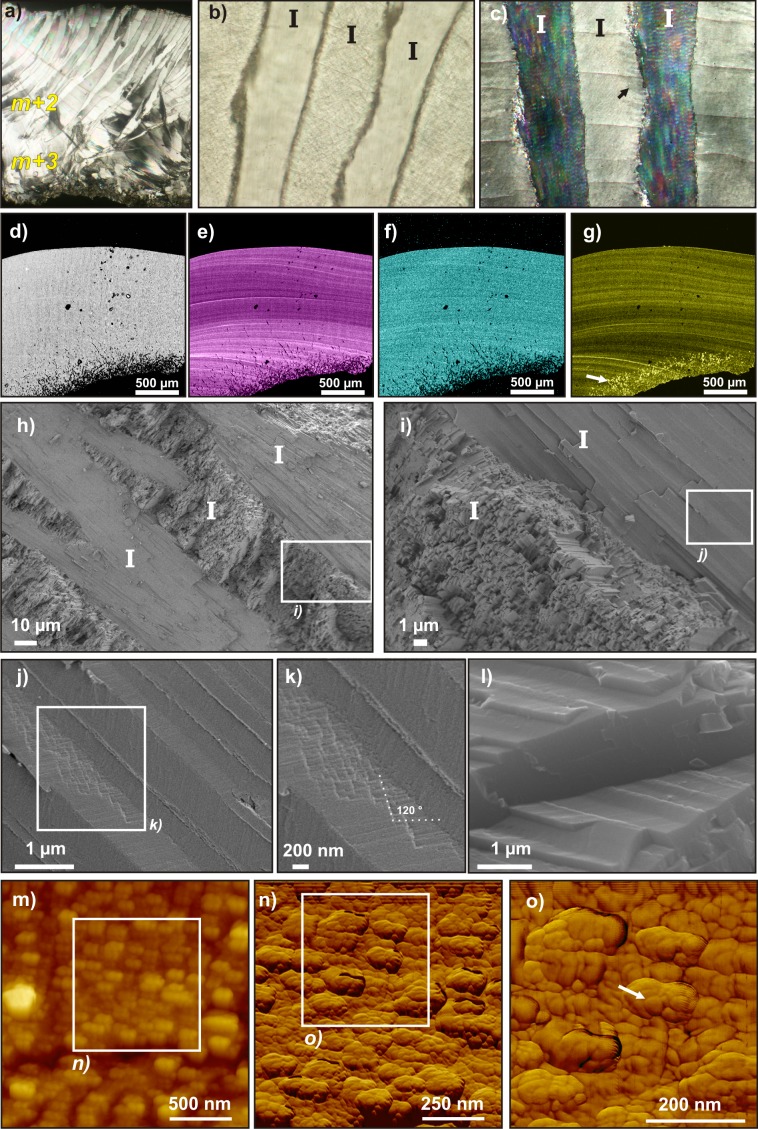
Microstructural organization of fossil *Patella* sp. calcite crossed-foliated outer layers. **a)** PLM view (polarized and analyzed light) of a radial thin section. Outer surface is on bottom. **b)** PLM view (polarized light) of several consecutive 1^*st*^ order lamellae (I). **c)** PLM view (polarized and analyzed light) of the same area, displaying the preserved alternate orientation one 1^*st*^ lamella on two. Black arrow marks a region of possible recrystallization. **d-g)** Electron micropobe maps. **d)** Backscattered image of the scanned area, showing the alternate 1^*st*^ order lamellae. **e)** Distribution of Mg content, strongly marking the growth layering. **f)** Distribution of Sr content, faintly marking the growth layering. **g)** Distribution of S content, enriched along growth layers but also within the canaliculi left by microboring organisms (white arrow). **h-k)** FEG-SEM images of a radial, unetched, freshly broken section. **h)** 1^*st*^ order lamellae (I). **i)** Limit between two 1^*st*^ order lamellae (I), showing the change of orientation of its constituting 3^*rd*^order slats. **j)** Surface view of 3^*rd*^ order slats forming one second order row. **k)** Surface view of one 3^*rd*^order units, displaying angular-shaped sub-units. **l)** SEM image of abnormally thick 2^*nd*^ order units, probably composed of several fused lamellae. **m-o)** AFM scans. **m)**Height image within a 1^*st*^ order lamella. **n)** Phase images of its constituting 3^*nd*^ order rods. **o)** Phase image of the 3^*nd*^ order rods, showing that they are composed of ovoid sub-units (white arrow).

The m+3 layer is thicker than in modern specimens and the transition from the radial to the concentric crossed-foliated pattern is progressive (Fig 5a). In polarized light, the first order lamellae are well marked, but the regular growth increments observed in modern *Patella vulgata* are not (Fig 5b). Instead, fractures irregularly divide first order lamellae, along the direction where growth increments should be observed, and they are not interconnected to adjacent lamellae (Fig 5c). However, the alternate orientation of first order lamellae is preserved, as seen in polarized and analyzed light (Fig 5c), and even their second and third order patterns can be observed, and exactly match the modern shell features (Fig 4c). For some given positions of the crossed-polarizers, faint crystallographic dis-orientations are visible close to first order lamellae borders (black arrows). These are not structure-related, and may correspond to regions of recrystallization of the mineral phase at a very fine scale.

On electron microprobe maps, minor element distributions are very similar to modern *Patella vulgata*shell distributions: they follow the growth layering well, with strong contrasts in Mg and S content (Fig 5e and 5g) and slight differences in Sr content (Fig 5f). Canaliculi left by microboring organisms were observed on the outer surface of the shell. Such canaliculi can cut quite deep into the shell layer (Fig 5d, 5e, and 5f) and appear as zones depleted in Mg and Sr. Quite surprisingly, they are still rich in S, therefore probably still filled with organic components from microborers. As in the modern shell, Ba and Mn contents are low and evenly distributed, so their maps are not shown here.

SEM views highlight the generally well-preserved and easily recognizable crossed-foliated organization at all microstructural levels: first order lamellae are clearly delimited (Fig 5h), composed of third order slats dipping in opposite directions (Fig 5i). In a few places however, some uncommon features can be observed: close to the boundary of first order lamellae (Fig 5j), the fracture of the shell highlights some angular-shaped constituents within third order rods, with a 120° angle typical of rhombohedral idiomorph calcite crystals (Fig 5k). The complex and fine pattern observed on the surface of second order lamellae in the modern specimen is missing, as are the limits separating third order slats. Moreover, the second order lamellae often appear to be unusually thick, as if fused together (Fig 5l). Etching or staining using OsO_4_ vapor remained unsuccessful.

On AFM scans, third order rods can be easily identified (Fig 5m), but the second order lamellae are not so obvious. In particular, the strong visco-elasticity contrast found at the boundary of second order lamellae in modern *Patella vulgata,*is not visible in phase images on the fossil specimen (Fig 5n), where no clear separation can be seen between second order rows. Ovoid sub-units are nonetheless still present, irregularly shaped and make up the third order units (Fig 5m).

### *In situ* characterization of organic components

#### Modern *Patella vulgata*

**Fig 6:**
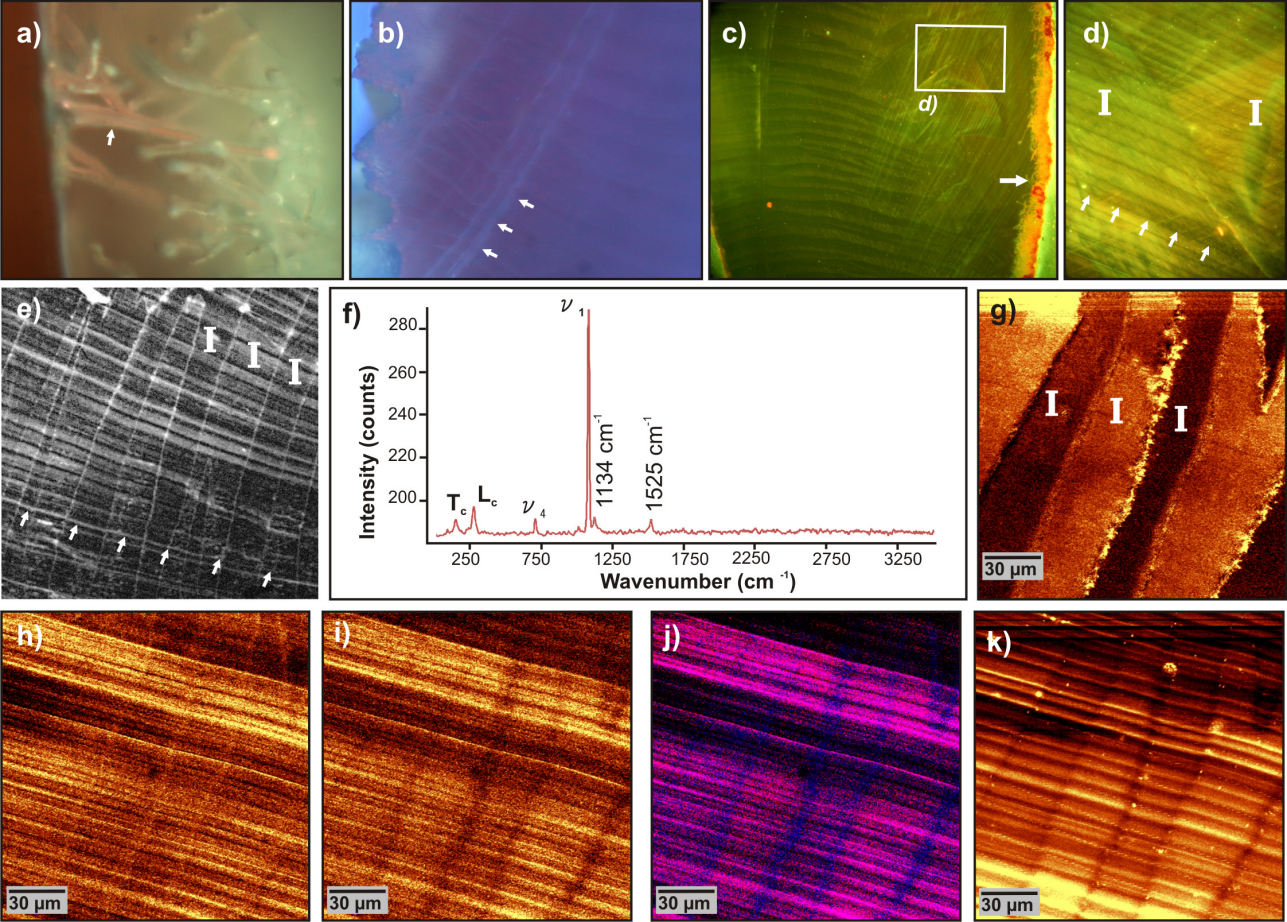
Epifluorescence and laser scanning microscopies of modern *Patella vulgata* calcite crossed-foliated outer layer. **a)** Canaliculi left by microboring organisms (white arrow) on the outer side of the shell (UV excitation). **b)** Inner border of the m+2 layer (UV excitation). **c)** Acridine orange-stained sample (blue excitation). White arrow marks micro-borer canaliculi on the outer side. **d)** Same as, focused in the m+3 layer. **e)** Zoom between two 1^*st*^ order lamellae (I), white arrows mark a growth increment. **e)** Confocal image of natural fluorescence of the sample under 488 nm excitation. White arrows mark a growth layer. **f)** Raman spectra extracted from following scan. **g)**Confocal Raman map showing the distribution of the ratio of calcite peaks L_*c*_ (librational mode, 282 cm^−1^) / *ν*_1_ (symmetric stretch, internal mode, 1085 cm^−1^), which highlights changes of crystallographic orientations of calcite between 1^*st*^ order lamellae (I). **h)** Map of the distribution of 1134 cm^−1^ band of polyenes. **i)** Map of the distribution of 1525 cm^−1^ band of polyenes. **j)** Composite map of i) (in blue) and j) (in red) showing anti- and co-localization of polyenic molecules. **(k)** Map showing the intensity distribution of the background intensity (between 2400 cm^−1^ and 2500 cm^−1^) related to the fluorescence of the sample.

The canaliculi left by microboring organisms, attacking the outer sides of shells, display a fluorescence response when subjected to UV excitation (365 nm, Fig 6a). The shell itself, however, does not display a strong epifluorescence under the same UV excitation, except for some well-marked growth increments (Fig 6b). The acridine orange staining procedure works well and its binding to specific organic molecules reveals microstructural features (435 nm excitation) not visible with autofluorescence only. On the outer side of the shell, the canaliculi formed by microboring organisms are strongly marked by an orange-red fluorescence under blue excitation, while the crossed-foliated pattern of layers m+3 and m+2 is marked by a fainter green (sometime tending towards orange) fluorescence color (Fig 6c). This highlights the alternate organization of first order lamellae (Fig 6c), but also their third order rods dipping in opposite directions (Fig 6d). Growth increments are also particularly marked in the m+3 layer (Fig 6c), and are perfectly continuous through crossed-foliated lamellae (Fig 6d).

On unstained samples, growth increments become visible when their autofluorescence is induced by excitation with a blue laser (488 nm) under confocal laser scanning microscope (Fig 6e); at this wavelength, a strong fluorescence from boundaries of the first order lamellae can also be observed. This fluorescence appears to correspond to seemingly continuous organic sheets, or membranes (Fig 6e).

Using confocal Raman microscopy, calcite is confirmed as the only mineral phase (Fig 6g). However, the Raman spectra also shows two peaks (at 1134 cm^−1^ and 1525 cm^−1^) (Fig 6f), which are not related to the calcite spectra, but characteristic of Resonance Raman (RR) spectra (respectively C-C and C = C stretching peaks), from molecules presenting a central polyenic chain (as in *β*-carotene). Many of the pigments widely found in the colored parts of mollusk shells are polyenes, either isolated or bound to other molecules, and can be accurately documented using RR Spectroscopy [71]. Indeed, due to the resonance coupling effect with the laser wavelength, even a small amount of pigments in the sample (down to 10^−8^ M) [72] can induce detectable peaks, leading to some structural characterizations of the molecules [73]. Here, these polyene peaks can be finely correlated to the microstructural pattern using the ratio of calcite peaks L_*c*_/*ν*_1_, as this ratio is related to the crystallographic orientation of the lattice [74]. The mapping of this peak ratio highlights the alternate shift of crystallographic orientations between consecutive first order lamellae (Fig 6g). The distribution of polyene peaks at 1134 cm^−1^ and 1525 cm^−1^ marks the relative distribution of polyenic molecules richer in C-C and C = C bonds respectively. These molecules are both distributed in growth increments, perpendicular to first order lamellae (Fig 6h and 6i), reflecting the cyclic secretion of these compounds by the mantle during shell growth. A composite in false color of maps at 1134 cm^−1^ (in blue) and 1525 cm^−1^ (in red) shows the co-localization of these peaks within the growth layers and their anti-localization (only blue color) within the membranes between the first order lamellae (Fig 6j). The polyenic molecules present in the membranes therefore display a higher ratio of C-C / C = C bonds than the polyenes present in the growth layers. Following the method developed in [74, 75], a range of the spectrum without pronounced Raman peaks (the range between 2400 cm^−1^ and 2500 cm^−1^) is used to map the fluorescence distribution throughout the mapped area. This map reflects the distribution of all the organic molecules with an epifluorescence response to a 532 nm excitation laser light (Fig 6k). Their distribution also varies following the growth layering, but it does not match the distribution of polyenic molecules (Fig 6j).

#### Fossil *Patella* sp

**Fig 7:**
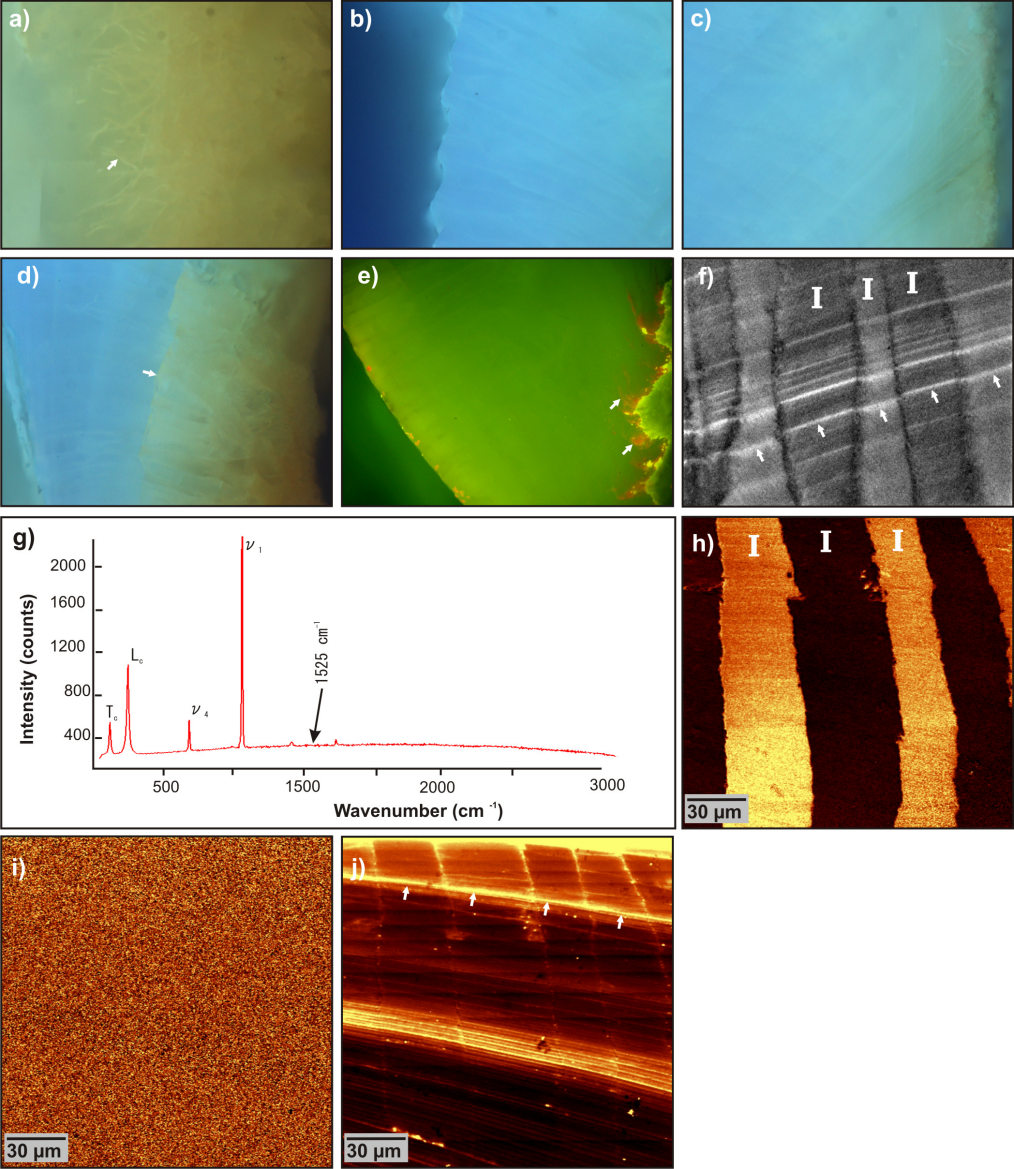
Epifluorescence and laser scanning microscopy of fossil *Patella sp.* calcite crossed-foliated outer layer. **a)** Canaliculi left by microboring organisms (white arrow) on the outer side of the shell (UV excitation). **b)** Inner border of the outer layer (UV excitation). **c-d)** Faint (c) and strong (d) changes of natural fluorescence hue of the shell at the contact with the sediment. **e)** Acridine orange-stained sample (blue excitation). white arrows mark some micro-borer canaliculi on the outer side. **f)** Confocal image of natural fluorescence of the sample under 488 nm excitation. White arrows mark a growth layer. **g)** Raman spectra extracted from previous scan. **h)**Confocal Raman map showing the distribution of the ratio of calcite peaks L_*c*_ (librational mode, 282 cm^−1^) / *ν*_1_ (symmetric stretch, internal mode, 1085 cm^−1^), which highlights changes of crystallographic orientations of calcite between 1^*st*^ order lamellae (I). **i)** Map of the distribution at the wavenumber that should correspond to 1134 cm^−1^ band of polyenes. **j)** Map showing the intensity distribution of the background intensity (between 2400 cm^−1^ and 2500 cm^−1^) related to the fluorescence of the sample.

As in modern *Patella vulgata*, the imprint of the canaliculi left by the microboring organisms can still be observed on the outer side of the shell under UV light (Fig 7a). But all the fossil shells display much stronger epifluorescence under UV light than the modern samples and hardly reveal any microstructural features (Fig 7b). Moreover, again under UV light, a brownish coloration of the outer side of the shell is often visible, sometimes limited to a thin layer that progressively disappears (Fig 7c), and sometimes affecting a thicker part of the shell with clear limits (Fig 7d). The acridine orange staining procedure remains unsuccessful for marking any microstructural feature, but still marks some of the microborer canaliculi on the outer side of the shell (Fig 7e). When excited with a blue laser (488 nm) under confocal laser scanning microscope, an autofluorescence response can be observed in some growth layers (they may be slightly more irregularly spaced than in modern samples). However, no fluorescence signal corresponds to the position of the membranes between first order lamellae (Fig 7f).

Raman confocal microscopy also confirms that calcite is the only mineral constituent (Fig 7g) and still displays an alternate shift of crystallographic orientations between consecutive first order lamellae (L_*c*_/*ν*_1_ peak ratio, Fig 7h). However, no peak corresponding to the polyenes can be found in the mean scan spectra (Fig 7g) nor in the map at 1525 cm^−1^ (or 1134 cm^−1^) (Fig 7i). The Raman spectra shows a higher background (Fig 7g), caused by a higher fluorescence of the sample. When mapped using background intensity between 2400 cm^−1^ and 2500 cm^−1^ (Fig 7j), the fluorescence signal still follows growth layering, although more irregularly than in modern *Patella vulgata*. Organic components seem to be better preserved in some areas than in others.

## Discussion

### Structural/Microstructural features

At a macroscopic scale, the fossil *Patella* sp. shells excavated from level 8 of El Haroura 2 Cave are very well preserved. The shells did not undergo the usual early diagenetic or biostratinomic processes that generally occur after the death of the animal [76]: major dissolutions/recrystallizations when the shell is left in sea water [77, 78], bioerosion [77, 79], or abrasion/fragmentation when the shells are exposed to sand blasting and/or rolled by waves on the shore [80–82]. This suggests that the mollusks were not gathered dead, as ¨empty shells ¨ on the beach. Thus, limpets were probably gathered live for consumption. The notches observed on some fossil shells (Fig 3i–3r) are similar to those obtained using tools on modern samples. This supports the hypothesis that Aterian groups gathered ¨fresh ¨ limpets, probably with adapted lithic tools.

Apart from marks of possible anthropogenic origin on the edges of some specimens, the shells are complete, and all the layers are preserved. The geological context of the cave is conducive to the good preservation of calcium carbonate remains, as the cave is carved into a calcarenite ridge (Fig 2a). Acidic rainwaters are therefore likely to be buffered when they percolate through the CaCO_3_ enclosing bedrock before they reach the shells.

At a microscopic scale, the crossed-foliated microstructure is also remarkably well preserved, down to a sub-micron scale. The first, second, and even third order lamellae morphologies and orientations of the fossil specimens exactly match those of modern samples (Fig 4 and Fig 6) in PLM (Fig 5a–5c), Raman L_*c*_/*ν*_1_ peak ratios (Fig 7h), and SEM views (Fig 5h–5i). Using AFM phase-lag signal, the third order units also appear to be composed of irregular, heterogeneous ovoid granules (Fig 5o), like those visible in modern *Patella vulgata*(Fig 4q). These granules are universally found within Ca-carbonate shells or skeletons: Mollusca shell layers [83], Scleractinia skeletons [84], Porifera calcareous structures [85, 86], Echinoida tests [52, 87], and Brachiopoda shells [88]. Their presence in a fossil shell emphasizes the fact that it has not undergone a major mineral phase modification. The distribution of Mg, Sr, and S minor element contents along growth layering is also very well preserved (Fig 5e, 5f and 5g), when compared to modern shells (Fig 4e, 4f and 4g).

A first approach would thus conclud that *Patella* sp. shells are mostly unaltered. But several indications point towards a possible diagenetic evolution. To the naked eye, all shells are depigmented (Fig 3e, 3i, 3j, 3n and 3o) and take on a coloration close to the sediment color. In PLM (Fig 5b), the highly regular growth increments found in modern shell (Fig 4b) have worn off and some areas display faint crystal dis-orientations close to the boundary of first order lamellae (Fig 5c). SEM views highlight uncommonly thick second order rows (Fig 5l) and even, in one spot, close to a boundary between first order lamellae, some angular-shaped components within third order rods (Fig 5k). These are highly unusual in a mollusk shell and may correspond to idiomorphic calcite crystal facets.

A more striking feature is the higher bulk fluorescence of the fossil *Patella* sp. compared to the modern shell exposed to a wide UV range excitation (mercury lamp) (Fig 7a–7d), also causing a significant background in Raman spectra (Fig 7g). Fluorescence of non-biogenic calcite is a common feature, and can be caused by many activators, resulting in a wide diversity of fluorescence colors. Shopov et al [89] listed more than 24 potential activators, the most commonly found being ions substituted within the crystal lattice (Mn^2+^, Fe^3+^, Pb^2+^, Ce^3+^, etc…) [90], or more complex ions (like uranyl ions), molecules (humic acids, fulvic acids, organic esters, etc…) or free radicals adsorbed within the crystal lattice. The brownish color observed under UV excitation, that sometimes gradually progresses inwards from the outer side of the shell (Fig 7c), and sometimes displays a clear limit (Fig 7d), could correspond to the migration of humic/fulvic acids or Fe/Mn issued from the sediment. The bulk, bright blue fluorescence (under UV excitation) is less clear and could be linked to any activator. Such strong fluorescence, in any case, implies a fundamental change in the calcite lattice. The lattice would have been modified to incorporate new ions in substitution and/or new ions or molecules adsorbed within the crystal defects. If such a mechanism occurred, even locally, in fossil *Patella* sp. shells, it happened while keeping the microstructural pattern preserved down to submicrometric scales. It would imply a (partial) recrystallization at very fine scale, smaller than the third order rod dimension, which is quite coherent with the observation of unusual angular units inside the rods.

Even though this recrystallization of the mineral phase is partial, it is prone to affect both isotopic and elemental ratios. It can therefore be expected that the classical palaeo-proxies based on such ratios would be affected. The same applies to dating attempts using radio-isotope measurements on the bulk calcitic phase, as we do not know when recrystallization occurred. It would therefore be appropriate to exclude the use of these fossil *Patella* sp. shells for further palaeoenvironmental or geochronological investigations.

### Organic content

#### The disappearance of organic membranes

The question of the occurrence of structural membranes within inter-crossed mollusk shell layers is still under debate, and of the utmost importance, as they may play an active role in the formation of these microstructural patterns [91]. Structural membranes can also have major implications for the modalities of the taphonomic/diagenetic evolution of these mollusk shell layers. The presence of sheets between second and/or third order lamellae in crossed-lamellar aragonite has long been reported in some bivalve shells, such as Tridacnidae [64], Glycymerididae,Cardiidae or Corbiculidae [92]. In other cases, these sheets are not reported, such as for the gastropod *Lottia kogamogai* (Patellogastropoda, Lottiidae) [93]. As for the boundaries between adjacent first order lamellae in crossed-lamellar microstructure, some authors consider that there are no membranes [64]. However, some indications suggest the presence of a non-continuous organic mesh in-between [74, 91]. Moreover, it has been shown that organic macromolecules extracted from limpet shells does impact the nucleation and the growth of aragonite crystals formed during *in vitro* experiments [94]. It is therefore important to validate the presence (or absence) of organic meshes or membranes structuring the crossed-lamellar microstructure, as the molecules forming these intricate organic patterns could be actively involved in the formation of such a complex 3D architecture.

In this respect, our finding of very similar membranes in crossed-foliated layers of modern *Patella vulgata* shells is quite consistent, as this structure is basically the calcitic counterpart of crossed-lamellar. An organic framework is indeed observed, separating third order slats within a second order row (Fig 4k–4m), and second order rows from each other (Fig 4p–4q). Moreover, seemingly continuous membranes are also visible at the boundaries of first order lamellae, but were only observed with scanning laser microscopy (Fig 6e–6h), and not on SEM views (Fig 4i). The membranes display strong autofluorescence under 488 nm excitation, and Resonance Raman maps indicate that they contain small amounts of polyenes (Fig 6j).

No similar framework of organic sheets or meshes is found in fossil *Patella* sp. shell, nor between first order (Fig 7f and 7i), second order (Fig 5n–5o) or third order (Fig 5j) lamellae. The disappearance of these structuring organic membranes is a first step in the taphonomic evolution of the shell. This can easily be explained by the fact that the molecules composing these pure organic meshes are not strongly linked to the mineral phase, unlike the organic matrix located within the mineral phase. This matrix is incorporated within the crystal at a molecular level [55]. The meshes are therefore more easily destabilized and liable to disappear first during fossilization processes such as hydrolysis, etc. The very classical example of *Pinna* illustrates this mechanism: the early degradation of the organic sheath surrounding the prisms leads to their dissociation and to the degradation of the prismatic layer. This process can occur very soon after the death of the animal and often only leaves the nacreous layer as fossil remain (in fact, this early diagenetic process even begins during the animal’s lifetime) [95].

Here, the degradation of the organic network can be correlated with the diagenetic evolution of the mineral phase. The disappearance of organic molecules separating second order rows would enable the observed fusing of several second order rows together (Fig 5l). Some modifications only tend to be visible close to the boundaries between first order lamellae, such as localized crystal dis-orientation spots in PLM (Fig 5c), or unusual angular-shaped components of third order rods (Fig 5k), whereas the bulk of the lamellae is better preserved (Fig 5n–5o). They could therefore concur with the disappearance of organic sheets separating first order rows.

#### Alteration/preservation of organic molecules

Membranes are not the only organic components affected. Whereas the depigmentation of the shell is visible to the naked eye, the disappearance of polyenic molecules distributed in the growth increments is confirmed at the micrometer scale (Fig 7i). The polyenic molecules represent a small percentage of the total organic content of the shell: indeed in a modern shell, their distribution does not match the distribution of fluorescent molecules (Fig 6k). However, Raman spectroscopy allows us to detect them with high sensitivity and these compounds therefore represent valuable markers to monitor the diagenetic evolution of fossil specimens.

The organic molecules that interact with acridine orange and were distributed in growth increments/third order rods in modern samples (Fig 6d), were probably degraded as no acridine fluorescence could be detected in the fossil shell. The interpretation of the fluorescent color caused by acridine orange staining is not straightforward. It is a cationic dye, which in solution emits in the green region (533 nm) when in monomer form, and in the red region (656 nm) in polymer form [96], the shift being caused by a dye-dye coupling effect. The emission color therefore depends on the distance between the stained molecules [97]. When acridine molecules are bound to neighboring target molecules, or neighboring sites on the same molecules, they display an orange-red fluorescence; when bound to isolated compounds, so that the dye molecules are weakly or not coupled with one another, the fluorescence is green [98]. Acridine orange is nonetheless commonly used [97, 99] as a dye for various compounds displaying available negative binding sites (such as glycoproteins) [100]. Here, due to the complexity of the organic matrix composition, we cannot draw conclusions on the characterization of the molecules stained by this protocol. A wide spectrum of fluorescence (green, orange, and red) is observed. But these organic compounds were nonetheless sufficiently modified or even completely disappeared during the taphonomic evolution of the fossil *Patella* sp. shell, so that they no longer bind acridine orange molecules.

A substantial fraction of the organic matrix is preserved and still displays autofluorescence under laser excitation (488 or 532 nm). These molecules are preserved *in situ*, still distributed in some well-marked growth layers, confirming that the organic corteges underwent differential diagenesis. But, as shown using the autofluorescence of organic compounds in confocal or CRM, many growth layers have been worn away. Any sclerochronological studies based on internal growth layering (daily/sub-daily growth increments) detected by means of organic components (fluorescence, etchings, etc…) are therefore prone to biases; the differential diagenesis of the organic matrix also rules out the use of the bulk biochemical composition of these fossil *Patella* sp. shells as palaeo-proxies.

#### Mollusks and human behavior

MP/MSA (Aterian) groups seem to have occupied the Témara caves ¨intensively¨ during the isotopic stage 5 (120-75 ka BP), undoubtedly in connection with periods of high sea levels when the caves where near the shore, as is the case today [17]. This location would have facilitated the exploitation of mollusks. Fresh limpets gathering would be confirmed at El Harhoura 2 Cave by the presence of notches and the good preservation state of limpet shells (no dissolution/recrystallization, no bioerosion and no abrasion/fragmentation aspects). However, notches have also been observed on Patellidae used as tool in Asturia dating from the Magdalenian [101]. In absence of micro-wear analyses (as it was made by Cuenca et al [101]), it is impossible to exclude the possibility that the *Patella* sp. shells from El Harhoura 2 have also been used as tools. Thus these notches may result both from their collecting and/or their use as tool. Anyway, these notches on fossil specimens are morphologically very similar to those obtained by collecting modern *Patella vulgata* with a knife.

Furthermore, the good preservation of limpet shells indicates that a very short period of time occurred between the gathering of the mollusks on the seashore and their deposition and burying in the cave. In addition, this preservation suggests that the sediment of level 8 is a favorable local micro-environment for mollusk shell conservation until the present time, unlike other archaeological sites, such as Die Kelder Cave [102]. Thus, at El Harhoura 2, limpet shell preservation allows us to document human behavior. Aterian groups of El Harhoura 2 (level 8) seem to have focused their selection on Patellidae, but it remains to be established whether these taxa were chosen among others, or whether they were the main available taxa in the local environment. The reconstruction of the geomorphology of the local coast is in progress, in order to identify coastal morphology during the cave occupations. The example of the neighboring cave of El Mnasra (unit 8, also dated to OIS 5) shows that the Aterian populations of Témara have exploited varied terrestrial (diverse ungulates and tortoises) and marine (mollusks) resources [11, 17, 27]. In addition, different activities were carried out on-site (lithic production, butchery, terrestrial faunal and mollusk consumption, use of fire, bone tools, *Nassarius* beads, and worked pigments). These results are similar to those from Southern Africa, such as Pinnacle Point Cave [33–35], Yserkfontein [36] or Blombos [103–105].

The apparent good preservation state of the limpet shells from El Harhoura 2 could suggest that they were well suited to further studies focusing on human behavior. Sclerochronological investigations, for example, could lead to the identification of occupation seasons at the site and help to establish a mobility model for MP/MSA groups in North Africa; a question still under debate. Indeed C. Marean [33, 34] suggests that the first southern AMH planned their visits to littoral areas according to tidal cycles, in turn dependent on lunar cycles. Thus, during spring tides MSA groups could have exploited the shore for a longer period without danger from waves. But the faint diagenetic degradations evidenced in the present study tend to bias such investigations and severely hinder these perspectives.

## Conclusion

Crossed-foliated layers of *Patella* sp. shells retrieved from level 8 of El Harhoura 2 Cave (MP/MSA, between 92 +11/-9 and 106.7 +/- 6.6 ka BP) are well preserved. Their microstructural patterns are conserved down to sub-micrometric scales, as is the layered distribution of several minor elements along growth increments (Mg, Sr, S). However, the shells present signs of alteration in that faint taphonomic degradations of both the mineral and organic components are observed. The most striking feature is the disappearance of organic envelopes or sheets between crossed-foliated lamellae. This is undoubtedly correlated to a faint diagenetic evolution of the mineral units close to the envelopes, which have partially recrystallized, but still retain their microstructural pattern. Along with these envelopes, some other organic molecules disappeared (or were degraded to the point where they cannot be detected anymore through spectroscopic or staining characterizations). A substantial fraction of the organic matrix is nonetheless preserved *in situ* and is distributed along some growth layers.

Given the age of the specimens, such a good preservation state is of course linked to the archaeological context: 1) human consumption implies that shells were gathered live on the seashore and did not roll on the shoreline or undergo the usual biostratinomic or early diagenetic processes in a marine environment. In addition, the notches on the limpet shells would confirm that they were gathered live and indicate that these mollusks were collected with adapted lithic tools. 2) The sedimentation in El Harhoura 2 Cave promotes good conditions for shell preservation, such as the buffering effect of the enclosing calcarenite on percolation waters, limiting substantial shell modification.

Our results thus provide a solid case-study for the early stages of the diagenetic evolution of crossed-foliated shell layers. Mechanisms occurring at fine microstructural scales are not often taken into account and the characterization of the early stages of degradation identified in this paper provide a good comparative basis for decrypting the diagenetic history of more heavily degraded specimens.

However, these results are not very promising for other future investigations based on *Patella* sp. shells from El Harhoura 2 Cave focusing on human behavior, or palaeo-environmental or geochronological reconstructions. As shown above, the confocal autofluorescence of organic compounds indicates that some growth layers have been worn away. This would evidently impact any sclerochronological studies based on internal growth layering (daily/sub-daily growth increments), which rely on organic components (fluorescence, etchings, etc.). Any bulk analysis of isotopic or minor element compositions should also be undertaken with considerable caution, as these proxies are prone to biases owing to the diagenetic state described above. The same applies to dating attempts using radio-isotope measurements on the bulk calcitic phase. Moreover, as the shell alterations described here are not limited to the outer surfaces, but affect the whole shell layer, none of the classical ¨cleaning¨ procedures, consisting of removing the outermost parts (for example by acidic etching), are likely to work. One possible approach would be the use of localized isotopic or minor element measurements with resolved techniques (SIMS, nanoSIMS). The stronger diagenetic effects should be localized at the border of the first order lamellae, so *in situ* measurements performed in the central part of the lamellae should be less impacted.

More generally, this study clearly illustrates the importance of establishing the preservation state of fossil shells through careful microstructural characterization, before assessing the viability of further specific investigations. *Patella* sp. shells from El Harhoura 2 Cave appear to be very well preserved, even down to the micrometer scale and the faint alterations documented here could very easily have gone unnoticed. Yet they would undoubtedly bias any proxy based on bulk elemental, (radio)-isotopic or biochemical composition.
